# Supplementary material for: FINDER: an automated software package to annotate eukaryotic genes from RNA-Seq data and associated protein sequences
Source: BMC Bioinformatics. 2021 Apr 20;22:205. doi: 10.1186/s12859-021-04120-9 (PMC8056616; doi:10.1186/s12859-021-04120-9)
Supplement: Supplementary file 1 — Additional file 1. Supplementary figures (S1–S9). [file 12859_2021_4120_MOESM1_ESM.pdf]

Figure S1

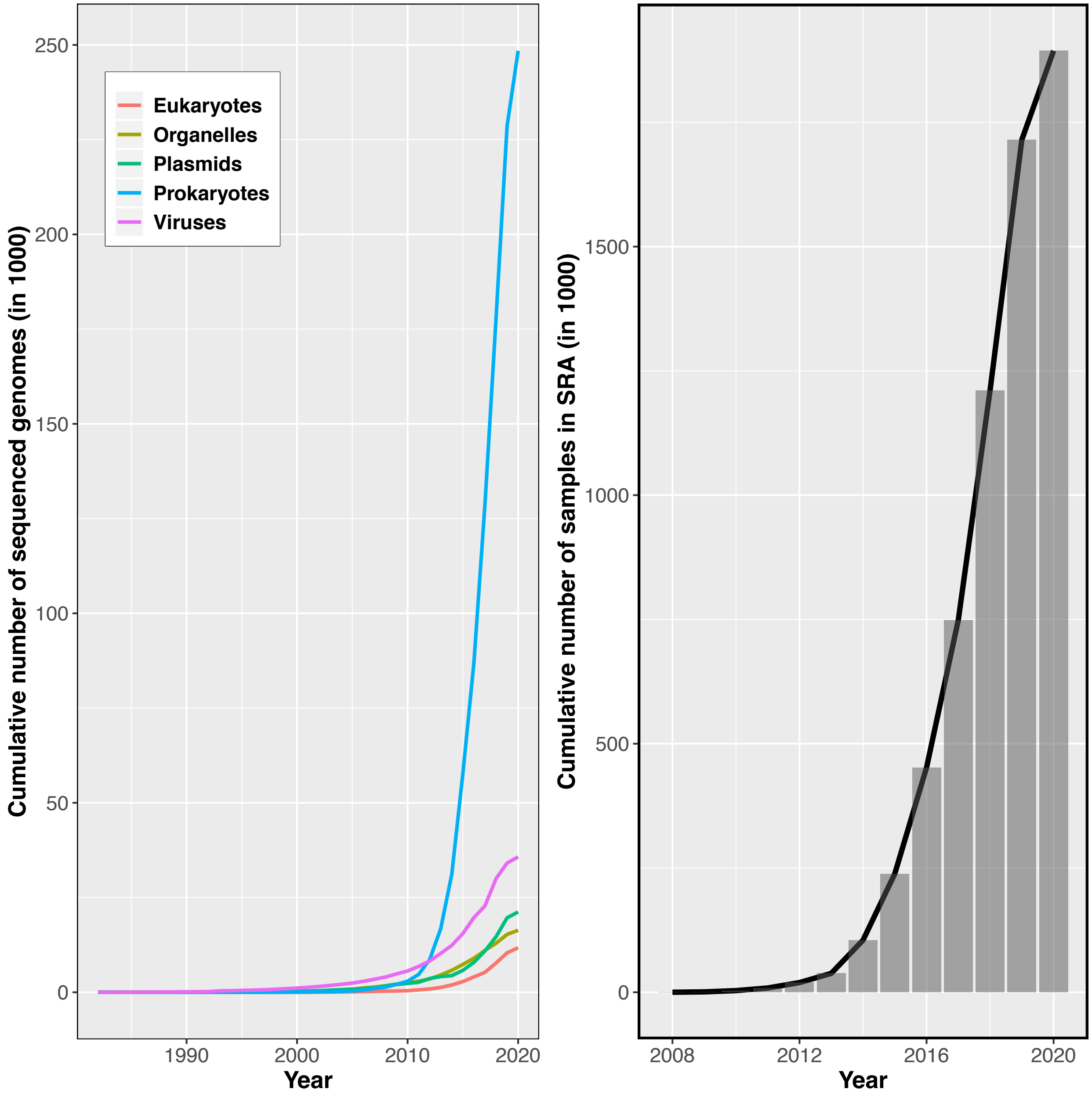

Fig. S1 Increase in the number of genomes and the number of sequenced samples over the course of the last decade

Figure S2

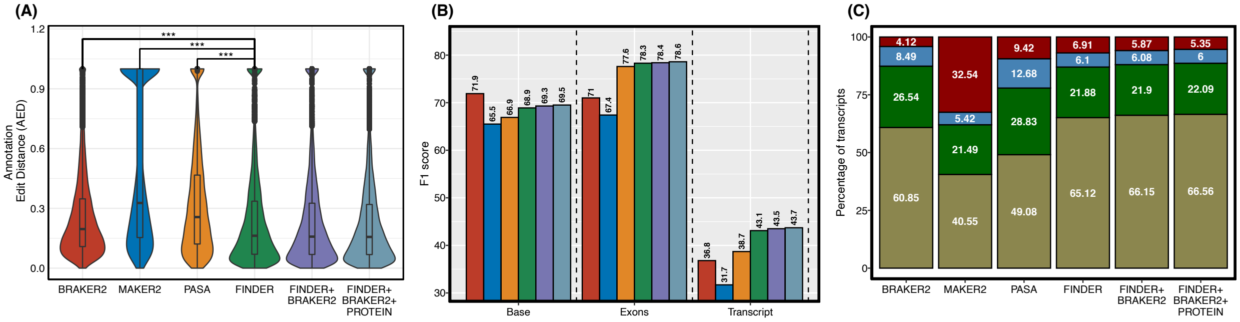

Fig. S2 Performance comparison of predicted annotations with reference annotations of ***Drosophila melanogaster*** (A) Distribution of AED scores (B) Bar plot of F1 scores (C) Stacked bar plot showing percentage of transcripts in each of the four groups of AEDs

Figure S3

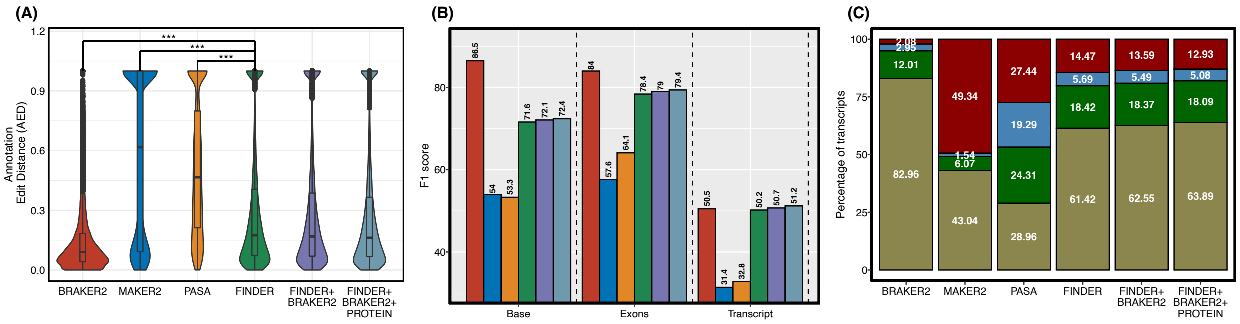

Fig. S3 Performance comparison of predicted annotations with reference annotations of ***Caenorhabditis elegans*** (A) Distribution of AED scores (B) Bar plot of F1 scores (C) Stacked bar plot showing percentage of transcripts in each of the four groups of AEDs

Figure S4

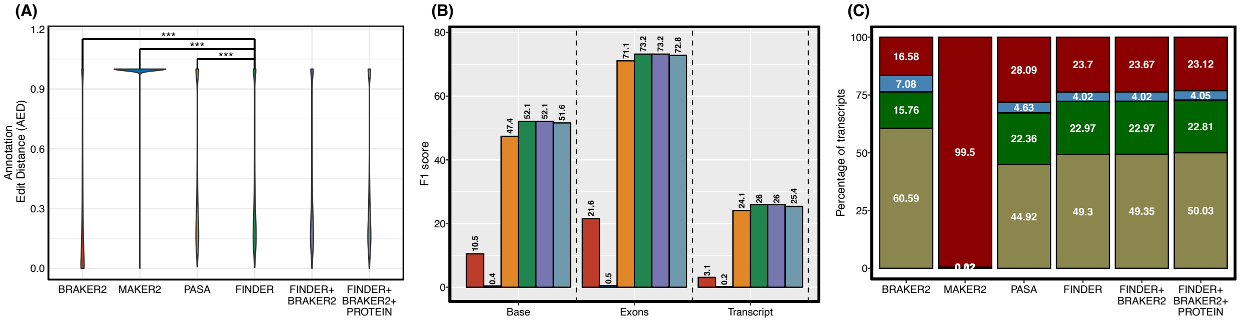

Fig. S4 Performance comparison of predicted annotations with reference annotations of ***Hordeum vulgare*** (A) Distribution of AED scores (B) Bar plot of F1 scores (C) Stacked bar plot showing percentage of transcripts in each of the four groups of AEDs

Figure S5

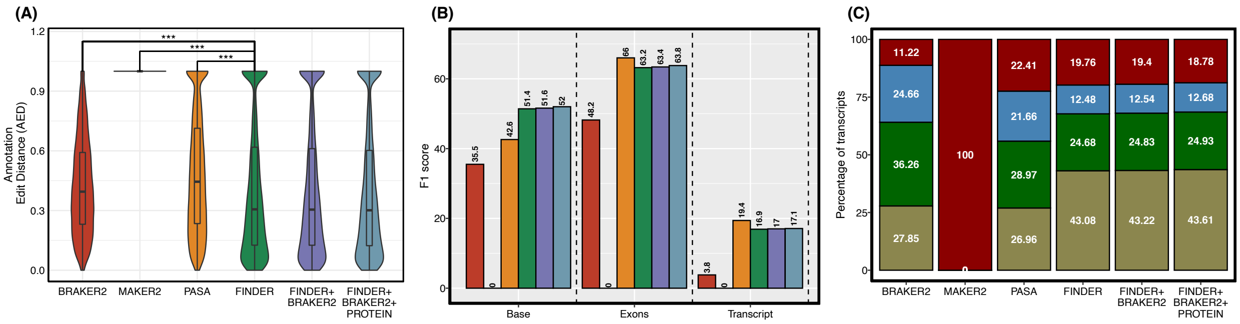

Fig. S5 Performance comparison of predicted annotations with reference annotations of ***Homo sapiens*** (A) Distribution of AED scores (B) Bar plot of F1 scores (C) Stacked bar plot showing percentage of transcripts in each of the four groups of AEDs

Figure S6

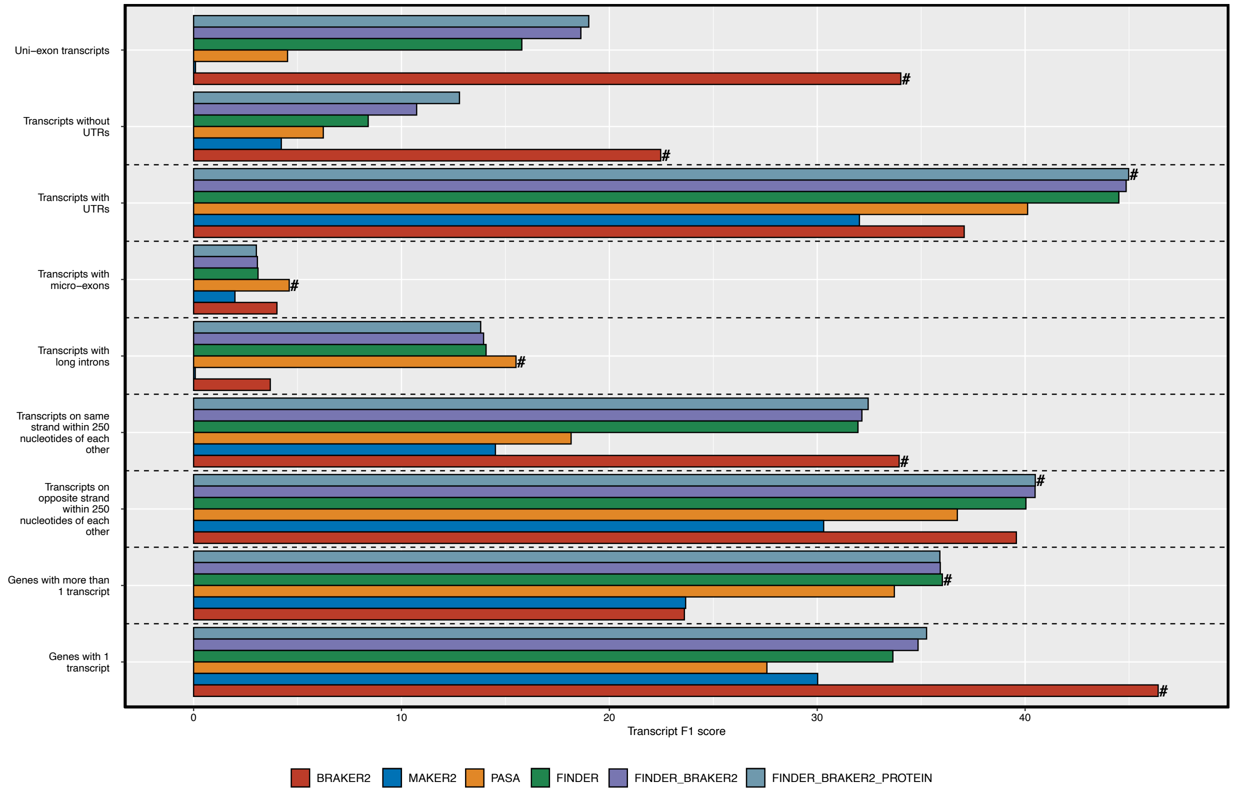

Fig. S6 Comparison of performance of FINDER with other gene annotation pipelines on different groups of genes in *Drosophila melanogaster*

Figure S7

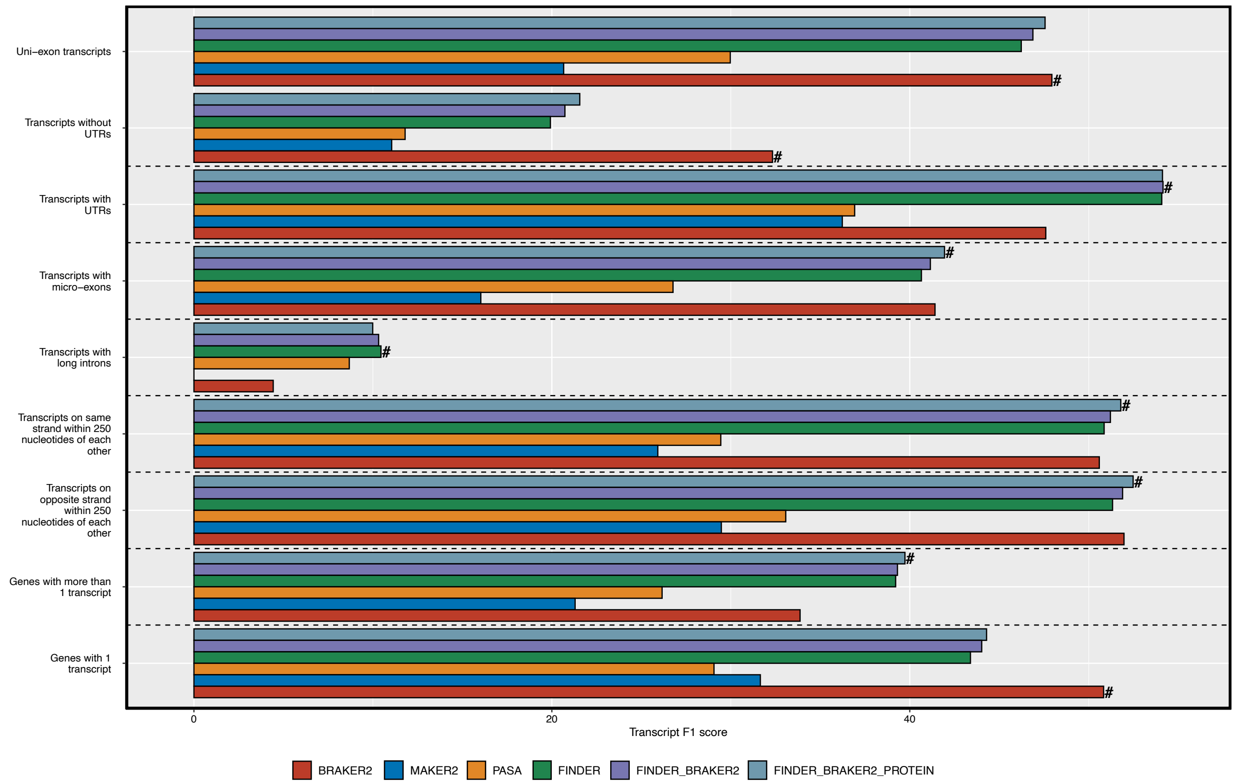

Fig. S7 Comparison of performance of FINDER with other gene annotation pipelines on different groups of genes in *Caenorhabditis elegans*

Figure S8

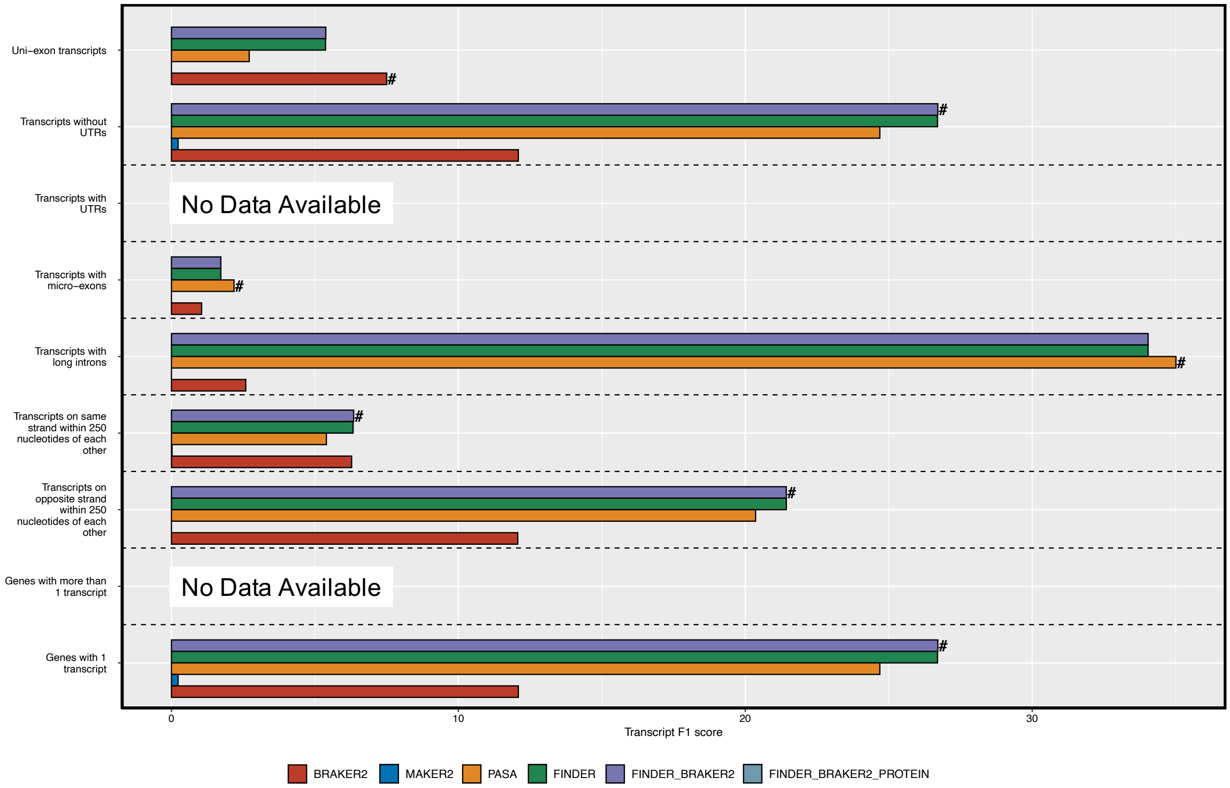

Fig. S8 Comparison of performance of FINDER with other gene annotation pipelines on different groups of genes in *Hordeum vulgare*

Figure S9

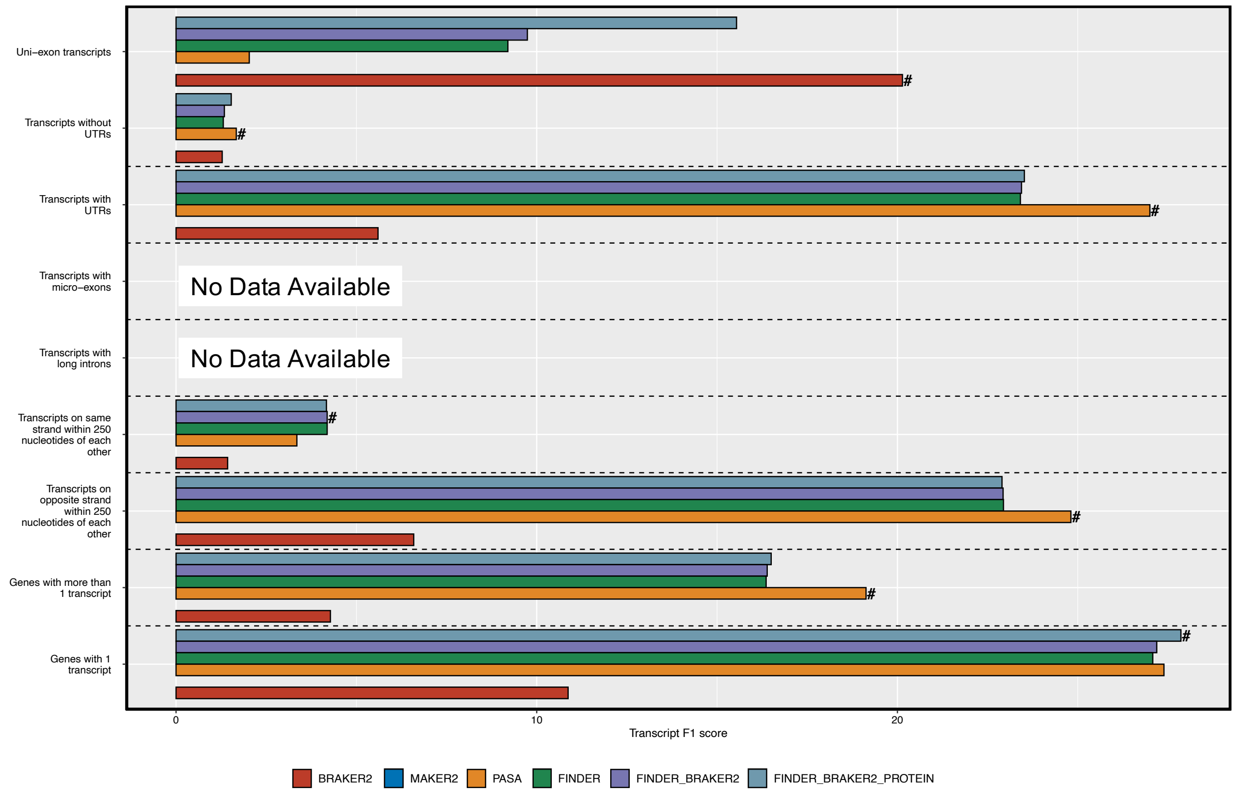

Fig. S9 Comparison of performance of FINDER with other gene annotation pipelines on different groups of genes in *Homo sapiens*
